# Hormonal associations in frontal fibrosing alopecia: A systematic review

**DOI:** 10.1016/j.jdin.2026.05.005

**Published:** 2026-05-12

**Authors:** Raymond Z. Ezzat, Li-Chi Chen, Meshi Paz, Caroline Kreytak, Maryanne M. Senna

**Affiliations:** a Department of Dermatology, Lahey Hospital & Medical Center, Burlington, Massachusetts; b Department of Dermatology, University of Massachusetts Medical Center, Worcester, Massachusetts; c Department of Dermatology, Harvard Medical School, Boston, Massachusetts

**Keywords:** androgens, endogenous hormones, exogenous hormones, framework, frontal fibrosing alopecia, hormone replacement therapy, hormones, HRT, OCP, oral contraceptive pills

*To the Editor:* Frontal fibrosing alopecia (FFA) is a primary cicatricial alopecia of uncertain etiology.^1^ Hormonal influences have long been proposed, yet prior studies are methodologically heterogeneous and difficult to interpret.^2^ We therefore conducted a systematic review of exogenous and endogenous hormonal factors in frontal fibrosing alopecia.

PubMed, Embase, and Web of Science databases were systematically searched through August 2025 (Supplementary eMethods, available via Mendeley at https://data.mendeley.com/preview/y487ff4mjm?a=5bf057a3-6545-4137-b81e-aa825e4dc18e). A total of 11 studies met the inclusion criteria (1948 FFA cases across 10 datasets and 1 electronic health record cohort involving 45,955 cases combining FFA with lichen planopilaris). Data screening and extraction were performed independently by 2 reviewers (R.Z.E., M.P.). Complete citations are available in Supplementary eReferences, available via Mendeley at https://data.mendeley.com/preview/y487ff4mjm?a=5bf057a3-6545-4137-b81e-aa825e4dc18e; study-level details are listed in Supplementary eTable I, available via Mendeley at https://data.mendeley.com/preview/y487ff4mjm?a=5bf057a3-6545-4137-b81e-aa825e4dc18e.

For endogenous hormones, small case–control and cross-sectional studies reported inconsistent abnormalities (eg, lower dehydroepiandrosterone sulfate, androstenedione, or FSH), but most values remained within reference ranges; no consistent endocrine profile emerged across studies.^3^, ^4^, ^5^

Several studies have reported associations between exogenous hormones and FFA, most often hormone replacement therapy (HRT) and oral contraceptives (OCPs). Moreno-Arrones et al found an association between HRT use and FFA in women (OR 1.76), while Singal et al identified links with HRT (OR 3.87) and OCPs (OR 2.92). Interpretation of these results is limited by exposure misclassification, outcome conflation, and incomplete understanding of hormone use. A gene–environment analysis revealed a CYP1B1 × OCP interaction (OR 1.90), suggesting pharmacogenomic susceptibility.

We conducted a random-effects meta-analysis of HRT and OCP exposure across 3 studies, which showed significant heterogeneity (I^2^ ≈ 93% and 98.5%) and limited interpretability. This pooled analysis highlights the limited comparability across the current literature, likely due to inconsistent case definitions, reliance on self-report, and the pooling of FFA with LPP.

Based on these current studies, endogenous hormone testing in FFA is inconsistent, and routine endocrine panels should not be ordered unless there is independent suspicion of endocrinopathy. Associations with HRT and OCPs are recurring but remain hypothesis-generating and confounded; reflex discontinuation of exogenous hormones is not supported, and decisions should be individualized.

To mitigate methodological heterogeneity, we propose a standardized set of data fields to enhance reporting and comparability in future research (Table I).

**Table I:** Proposed framework for future studies examining hormonal associations in frontal fibrosing alopecia

Domain	What to report
Population	FFA diagnostic criteria; method of diagnosis; biopsy confirmation if available; incident vs prevalent cases; and whether lichen planopilaris overlap was excluded or analyzed separately
Intervention/exposure	Hormone type or hormone measured; method of ascertainment, timing,; duration; and relevant details such as formulation or menopausal status
Comparator	Source and selection of controls; eligibility criteria; and matching or adjustment variables
Outcome	Definition of FFA outcome and how it was confirmed or coded

*FFA*, Frontal fibrosing alopecia.

In conclusion, current evidence does not establish a definitive hormonal cause for FFA. Endogenous findings remain inconsistent, and previously reported associations with exogenous hormones are not sufficiently strong to justify routine practice changes. More rigorous and standardized research is necessary to determine whether hormonal factors play a meaningful role in the development of FFA.

### Declaration of generative AI and AI-assisted technologies in the writing process

Not applicable.

## Conflicts of interest

None disclosed.
